# Overdone Sterilization

**Published:** 1896-02

**Authors:** 


### Overdone Sterilization.

There is a golden meaning in all things, and even the most salutary and sanitary practices can be carried to excess, says the *Journal of the American Medical Association* of December 14th. In what may be termed microbophobia, the extremes of caution as to the exposure to pathologic germs, there may be a danger on the other side. One may, in avoiding the Scylla of organic infection fall into the Charybdis of chemical poisoning, or starvation. These thoughts are suggested by a paper by Dr. Louis Starr in the December number of the *American Journal of the Medical Sciences*, in which he gives details of three cases of infantile scurvy due to the exclusive use of sterilized milk; and he further states that he has seen five other cases in consultation within eighteen months. He attributes the disease to the alterations that occur in the milk during the process of sterilization, especially in the lactalbumin which has its solubility diminished, and in the fat globules which coalesce with each other, and with some of the insoluble albuminous matters. All the cases recovered promptly on a diet of unsterilized milk, together with raw beef juice and orange juice and tonics.

